# Immune response of hibernating European bats to a fungal challenge

**DOI:** 10.1242/bio.046078

**Published:** 2019-10-24

**Authors:** Marcus Fritze, David Costantini, Jörns Fickel, Dana Wehner, Gábor Á. Czirják, Christian C. Voigt

**Affiliations:** 1 Leibniz Institute for Zoo and Wildlife Research, Alfred-Kowalke-Str. 17, 10315 Berlin, Germany; 2 Institute of Biology, Free University of Berlin, Takustr. 6, 14195 Berlin, Germany; 3 Unité Physiologie moléculaire et adaptation (PhyMA), Muséum National d'Histoire Naturelle, CNRS; CP32, 57 rue Cuvier 75005 Paris, France; 4 University of Potsdam, Institute for Biochemistry and Biology, Karl-Liebknecht-Str. 24-25, 14476 Potsdam, Germany

**Keywords:** Fungal challenge, Torpor, Body temperature, Zymosan, Acute phase response, Oxidative stress, *Myotis myotis*

## Abstract

Immunological responses of hibernating mammals are suppressed at low body temperatures, a possible explanation for the devastating effect of the white-nose syndrome on hibernating North American bats. However, European bats seem to cope well with the fungal causative agent of the disease. To better understand the immune response of hibernating bats, especially against fungal pathogens, we challenged European greater mouse-eared bats (*Myotis myotis*) by inoculating the fungal antigen zymosan. We monitored torpor patterns, immune gene expressions, different aspects of the acute phase response and plasma oxidative status markers, and compared them with sham-injected control animals at 30 min, 48 h and 96 h after inoculation. Torpor patterns, body temperatures, body masses, white blood cell counts, expression of immune genes, reactive oxygen metabolites and non-enzymatic antioxidant capacity did not differ between groups during the experiment. However, zymosan injected bats had significantly higher levels of haptoglobin than the control animals. Our results indicate that hibernating greater mouse-eared bats mount an inflammatory response to a fungal challenge, with only mild to negligible consequences for the energy budget of hibernation. Our study gives a first hint that hibernating European bats may have evolved a hibernation-adjusted immune response in order to balance the trade-off between competent pathogen elimination and a prudent energy-saving regime.

## Abstract (precis)

**Summary:** Our experimental immunological study on European bats provides new information on the functionality of the immune system in hibernation. For this we challenged bats with a fungal antigen and measured different immunological parameters.

## INTRODUCTION

Hibernation is a prolonged state of reduced metabolic activity and lowered body temperature in mammals and has evolved as a strategy to overcome winter periods with adverse weather conditions and low resource availability (Boyer and Barnes, 1999). During hibernation, all major physiological functions are reduced to save energy, which is generated by the oxidation of fatty acids from adipocytes (Florant, 1998). In small hibernating mammals, body temperature drops down to levels slightly above ambient temperatures for periods of 4–30 days, a stage called torpor (Bouma et al., 2010a). These torpor bouts are periodically interrupted by short arousal events during which animals increase their core body temperature and during which most of their physiological functions are restored (Hut et al., 2002).

In general, immune responses are energetically costly as they involve significant increases in the metabolic rate, which may ultimately deplete fat deposits during hibernation (Canale and Henry, 2011; Demas et al., 1997; Martin et al., 2003). Consequently, immune functions were found to be downregulated in hibernating mammals (Bouma et al., 2010a). Besides the general energy saving strategy associated with impaired immunity in hibernators, there is no need for an active immune system during torpor bouts because activities of most pathogens are also decreased at low temperatures. An exception is psychrophilic pathogens, especially some bacteria and fungi, which grow at low temperatures (Bižanov and Dobrokhotova, 2007; Brunet et al., 2018; Dempster et al., 1966). How hibernators cope with these potential pathogens when physiological functions are constrained is not yet fully understood. This question is even more important since the emergence of white-nose syndrome, a disease affecting hibernating North American bats after having contracted the psychrophilic fungus *Pseudogymnoascus destructans* (Frick et al., 2016; Wibbelt, 2018).

Pathogens that enter organisms are first recognized by the innate immune receptors, of which Toll-like receptors (TLRs) are highly relevant (Doan et al., 2013). Fungi are typically recognized by TLR2 and TLR4 on macrophages, as well as dectin-1, C-type lectins, and NLRP3 inflammasome (Calich et al., 2008; Salazar and Brown, 2018). Positive signals from these receptors expressed on the surface of sentinel immune cells such as macrophages, stimulate cells to secrete pro-inflammatory cytokines [e.g. interleukin 1β (IL-1β), interferon gamma (IFN-γ) and tumor necrosis factor α (TNF-α)], and anti-inflammatory [e.g. interleukin 10 (IL-10)] as well as inflammatory mediators like nitric oxide (NO) (Calich et al., 2008; Young et al., 2001). Under the influence of cytokines, the brain responds via modified behaviors (e.g. sickness behavior including anorexia, lethargy and sleepiness) and development of fever. During this acute phase, the bone marrow will increase white blood cell (WBC) production and the liver will initiate the synthesis and secretion of defense proteins, e.g. acute phase proteins such as haptoglobin, serum amyloid A or C-reactive protein (Tizard, 2008). All these modifications constitute the acute phase response, which is part of the innate immune defense known to be energetically costly (Lee, 2006). As a consequence of the acute phase response, immune cells release cytotoxic chemicals with pro-oxidant activity, which damage pathogens (Costantini, 2014; Schneeberger et al., 2013a; Sorci and Faivre, 2009). To avoid cell damage of the host, excess pro-oxidants need to be balanced by anti-oxidants. An imbalance between pro-oxidants and anti-oxidants leading to increased oxidative damage may occur as a consequence of an immune response (Costantini and Møller, 2009; Schneeberger et al., 2013a).

Compared with adaptive immunity, relatively little is known about innate immunity in hibernating mammals, specifically when challenged by an antigen or an infectious agent. *In vitro* studies on hibernating rodents report a decrease in pro-inflammatory cytokine production and phagocytosis in innate immune cells (Kandefer-Szerszeń, 1988; Novoselova et al., 2000). Moreover, golden-mantled ground squirrels (*Spermophilus lateralis*) challenged with bacterial lipopolysaccharide during a torpid phase developed fever only during the subsequent arousals, indicating that arousal events evolved, at least partly, in order to clear the pathogens accumulated during hibernation (Prendergast et al., 2002).

To better understand how hibernating European bats generally cope with fungal challenges, we followed aspects of the acute phase response over a 4 day period after challenging greater mouse-eared bats (*Myotis myotis*) with Zymosan, a homoglucan of n glucose molecules in ß-1,3-glycosidic linkage. Zymosan is a fungal antigen that activates macrophages via TLR2 and thus is regularly used in experimental studies to induce immune responses (Bastos-Pereira et al., 2018; Underhill, 2003; Volman et al., 2005). Besides the general interest to better characterize the acute phase response of hibernators against fungal pathogens, studies on European hibernating bats are important because of their apparent resistance against the white-nose syndrome causing agent *P. destructans* (Puechmaille et al., 2011; Wibbelt et al., 2013). Considering the energetic aspects of hibernation and the acute phase response and also the apparent resistance of this bat species against local fungal infections, we hypothesized that zymosan-challenged hibernating *M. myotis* will show an immunological response but with hibernation-related adjustments in the composition of the response in order to avoid activating processes that are energetically costly. Therefore, we predicted that zymosan-treated bats will not increase arousal frequencies and durations but will show a response dominated by acute-phase proteins. However, experimental bats may develop a fever response during arousals, and immune gene expressions in response to the zymosan challenge (Young et al., 2001). We expected the acute phase response in zymosan-treated bats to be characterized by an increase in haptoglobin and oxidative damage or antioxidant levels, but not in WBCs in order to save energy. If zymosan-treated bats do not bear higher energetic costs than their untreated conspecifics, then their loss in body mass during hibernation will not be higher than in bats of the control group.

## RESULTS

### Body condition

**Fig. 1.**
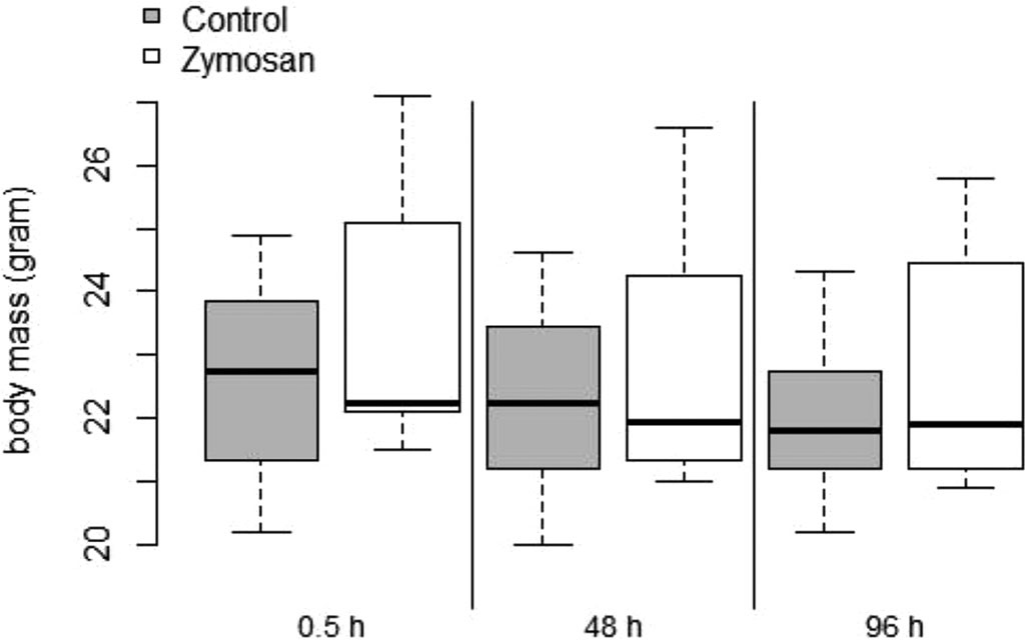
**Boxplot displaying the body masses of the zymosan-treated bats (white) and the bats of the control group (grey) during 5 experimental days.** Upper and lower borders of boxes indicate first and third quantiles, respectively, whiskers represent maximum and minimum, the median is shown as a solid horizontal line within boxes.

We observed changes in body mass (log_e_ transformed) in individuals across sampling days (SS=0.007, *F*=8.33, *P*=0.001) (Fig. 1). However, we neither detected significant effects in body mass changes between treatment group (SS<0.001, *F*=0.76, *P*=0.399), nor in the interaction between sampling day and treatment group (SS<0.001, *F*=0.34, *P*=0.713). Forearm length was also not a significant covariate (SS<0.001, *F*=0.41, *P*=0.536).

### Body temperature monitoring

**Fig. 2.**
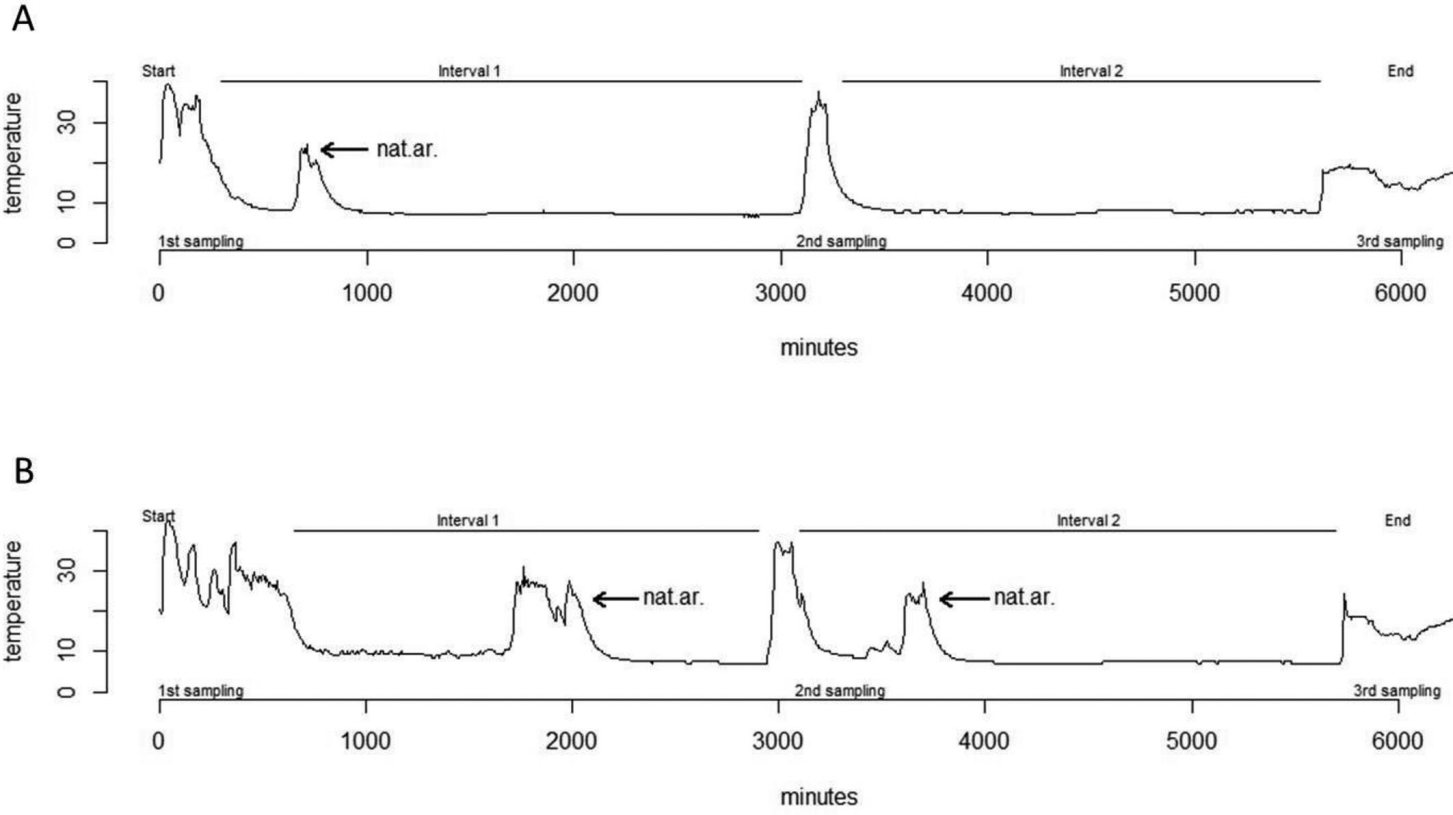
**Representative examples of a T_sk_ profile (°C) of a zymosan-treated bat (A) and a control bat (B).** The starting point (at 0 min) represents the arousal during the first injection and the first blood sampling on day 1; the second sampling took place 48 h (2880 min) after inoculation and the third sampling 96 h after the injection (5760 min). In the example shown here, the zymosan-treated bat (A) had one natural arousal (nat. ar.) in interval 1 (between the start of the experiment and the second sampling), but remained torpid in interval 2 (between the second and third sampling). The control bat (B) had two natural arousals, one in the first interval and one in the second.

**Fig. 3.**
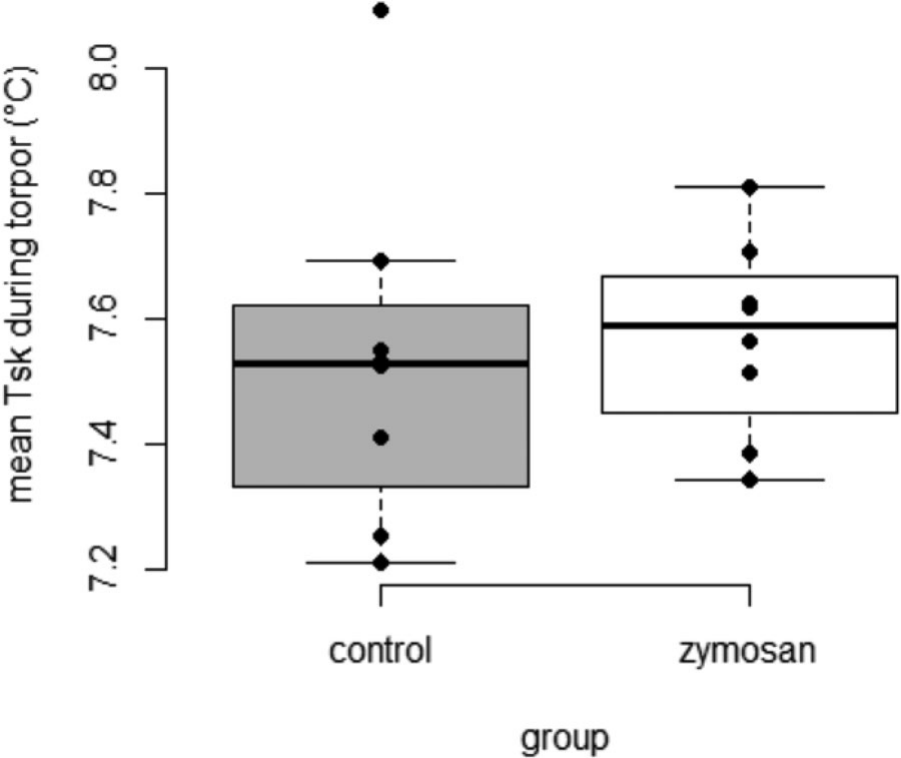
**Boxplot of the mean skin temperatures (mean T_sk_) during torpor (°C) of bats of the control (grey) and of the zymosan-treated group (white).** Upper and lower borders of boxes indicate first and third quantiles, respectively, whiskers represent maximum and minimum, solid horizontal line within boxes shows the median. Raw data are shown as solid circles.

Following the treatment-specific injections, all bats entered torpor, resulting in an average torpid skin temperature (T_sk_) of 7.6±0.5°C. There was no difference between treatment groups in the time required for aroused bats to decrease T_sk_ from euthermic state (T_sk_∼36.0°C) to the torpor state (T_sk_<10.5°C) (Ø 598 min, SS=1506, *F*=0.12, *P*=0.738, ANOVA). We then excluded all T_sk_ data from artificial arousal events from further analysis, i.e. we considered only T_sk_ data from the intervals between first and second blood sampling and between second and third blood sampling (Fig. 2) for further evaluating natural arousals. Data collected by temperature loggers amounted to about 1000 T_sk_ measurements per individual for analysis. The frequency of natural arousals did not vary between treatment groups (χ2=1.65, Df=3, *P*=0.649), i.e. in total, we observed five arousals in bats of the zymosan-treated group and four in the control group. The maximal T_sk_ during arousal periods did not differ between treatment groups (SS=1.190, *F*=0.07, *P*=0.806) suggesting no fever response after zymosan inoculation. We also did not find differences in the mean T_sk_ during arousal (SS=0.009, *F*=0.002, *P*=0.965) and mean T_sk_ during torpor (SS=0.005 *F*=0.11, *P*=0.748, Fig. 3) between treatment groups. The durations of arousals neither differed between treatment groups (SS=1650, *F*=0.15, *P*=0.712) nor between artificially induced and natural arousals (SS=0.072, *F*=0.01, *P*=0.928). On average, arousal events lasted for 190 min (Table 2) Also, the time required to increase body temperature by +10°C during rewarming was not different between treatment groups (SS=1.540, *F*=0.181, *P*=0.674). On average, bats required about 21 min to return to the euthermic state (see Table S1).

**Table 1.**
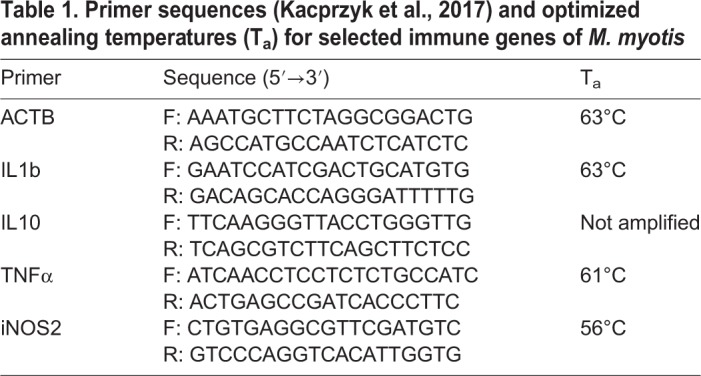
**Primer sequences (Kacprzyk et al., 2017) and optimized annealing temperatures (T_a_) for selected immune genes of *M. myotis***

**Table 2.**
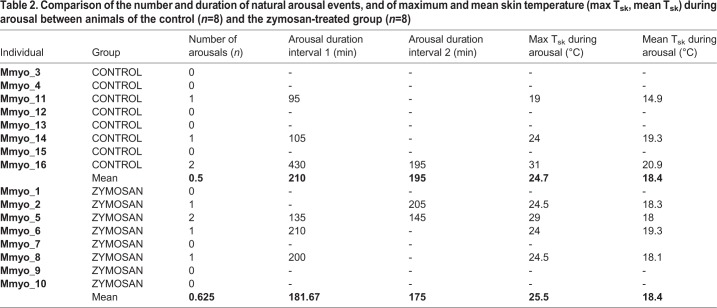
**Comparison of the number and duration of natural arousal events, and of maximum and mean skin temperature (max T_sk_, mean T_sk_) during arousal between animals of the control (*n*=8) and the zymosan-treated group (*n*=8)**

### Inflammatory gene expressions

**Fig. 4.**
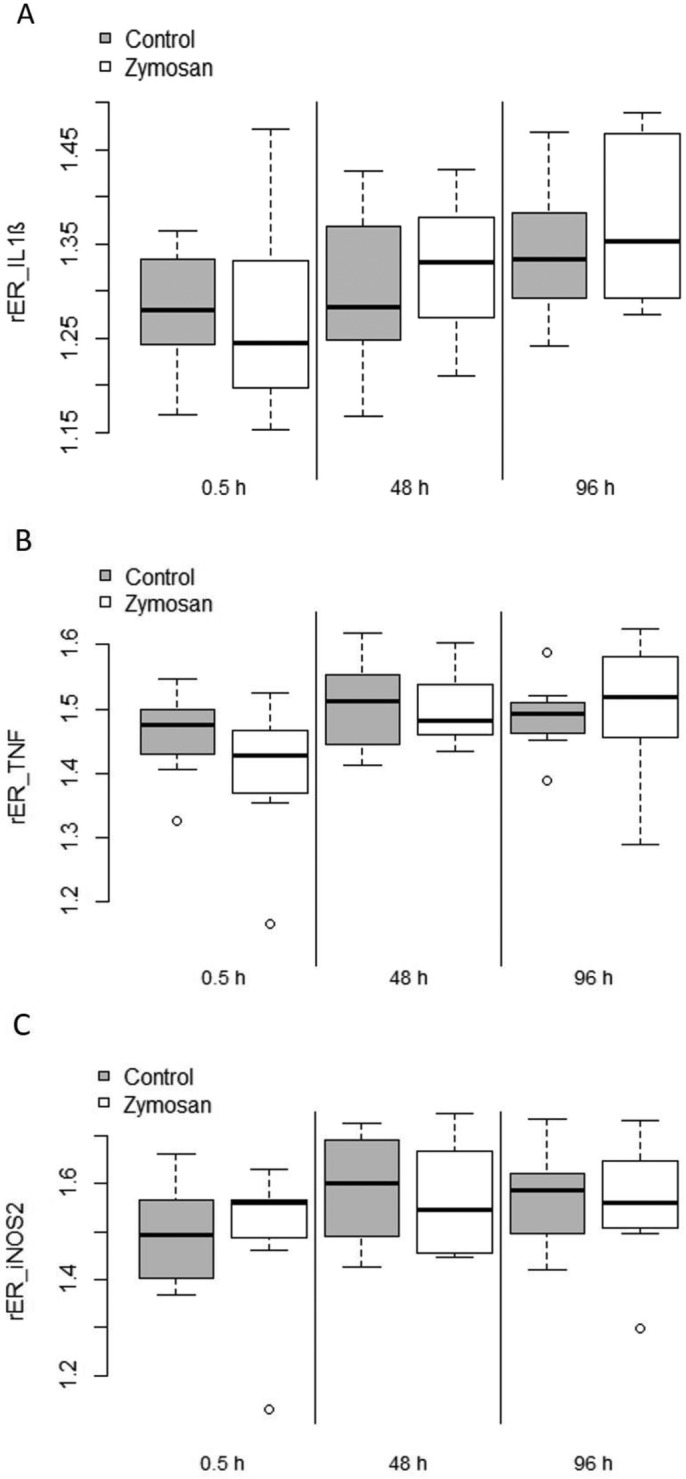
**Boxplots of relative immune gene expression ratios (rER) of three different genes: IL-1β (A), TNFα (B) and iNOS2 (C), measured at three sampling days for zymosan-treated bats (white) and bats of the control group (grey).** rERs are given in relation to the expression of ACTB. Upper and lower borders of boxes indicate first and third quantiles, respectively, whiskers represent maximum and minimum, solid horizontal line within boxes shows the median. Extreme values are shown as open circles.

We found significant increases in the gene expression levels of IL-1β across the sampling days in both treatment groups (SS=0.052, *F*=5.70, *P*=0.009). However, neither the treatment (SS<0.001, *F*=0.19, *P*=0.666) nor the interaction between treatment and sampling day (SS=0.004, *F*=0.43, *P*=0.655), nor the interaction between body mass and forearm lengths (SS=0.002, *F*=0.34, *P*=0.564) differed between treatment groups (Fig. 4). For iNOS2 expression, we also found significant increases across the sampling days in both groups (SS=0.11, *F*=3.87, *P*=0.031). Further, we detected a significant influence of the body condition on iNOS2 expression: mass (SS=0.067, *F*=4.91, *P*=0.034), forearm length (SS=0.066, *F*=4.85, *P*=0.034) and the interaction between body mass and forearm length (SS=0.064, *F*=4.70, *P*=0.037). However, there was no significant difference in iNOS2 expression between treatment groups (SS<0.001, *F*=0.037, *P*=0.848). TNFα expression significantly increased across the sampling days (SS=0.059, *F*=5.016, *P*=0.012). However, neither treatment group affiliation (SS=0.006, *F*=0.967, *P*=0.332), nor the interaction between treatment and day (SS=0.012, *F*=1.043, *P*=0.362) nor the body condition of animals (interaction mass and forearm: SS=0.011, *F*=1.915, *P*=0.175) had a significant effect on TNFα expression. IL-10 primers failed to amplify any fragment in a PCR and thus its expression could not be assessed in a qPCR. The mean of the RIN values of the RNA samples was 3.9 (SD=0.89). A full table with RIN values of all measured gene expressions is available in the supplementary material (Table S2).

### Haptoglobin

**Fig. 5.**
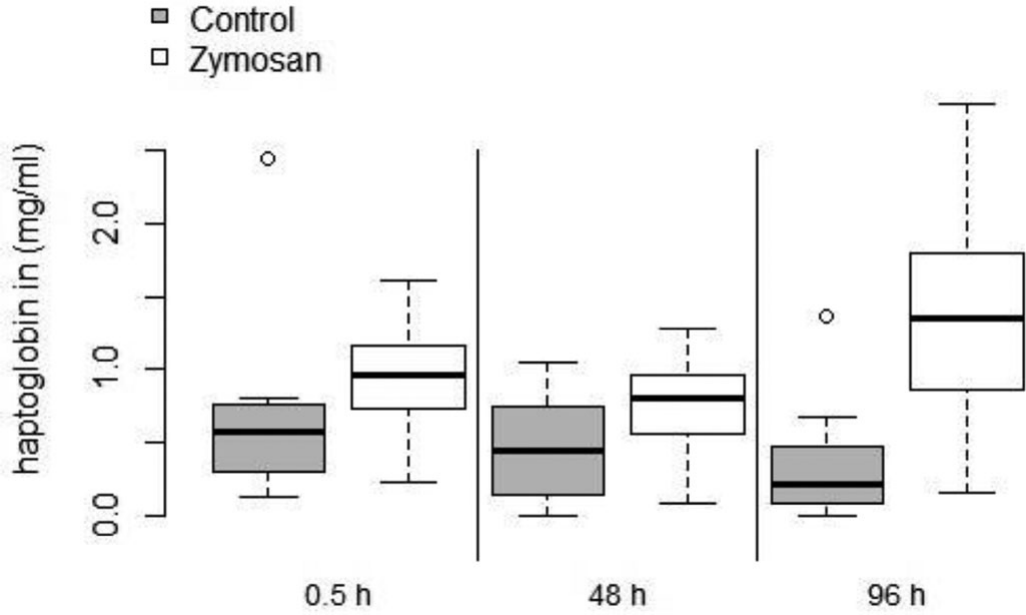
**Difference in haptoglobin concentration (mg/ml) in the blood of zymosan-treated bats (white) compared with control bats (grey) at three time points of the experiment: at 30 min, 48 h and 96 h after inoculation.** Upper and lower borders of boxes indicate first and third quantiles, respectively, whiskers represent maximum and minimum, solid horizontal line within boxes shows the median. Extreme values are shown as open circles.

We detected significant differences in haptoglobin levels (square root transformed) for the relationship of treatment group and sampling day (SS=0.531, *F*=3.84, *P*=0.033). Zymosan-treated bats had higher levels at 96 h post-injection compared with bats of the control group on the same day (SE=0.192, t=−3.154, *P*=0.046, Tukey adjusted) (Fig. 5). Forearm and body mass (interaction) had no influence on the haptoglobin levels (SS=0.023, *F*=0.34, *P*=0.568).

### Reactive oxygen metabolites (ROMs) and plasma non-enzymatic antioxidant barrier (OXY)

**Fig. 6.**
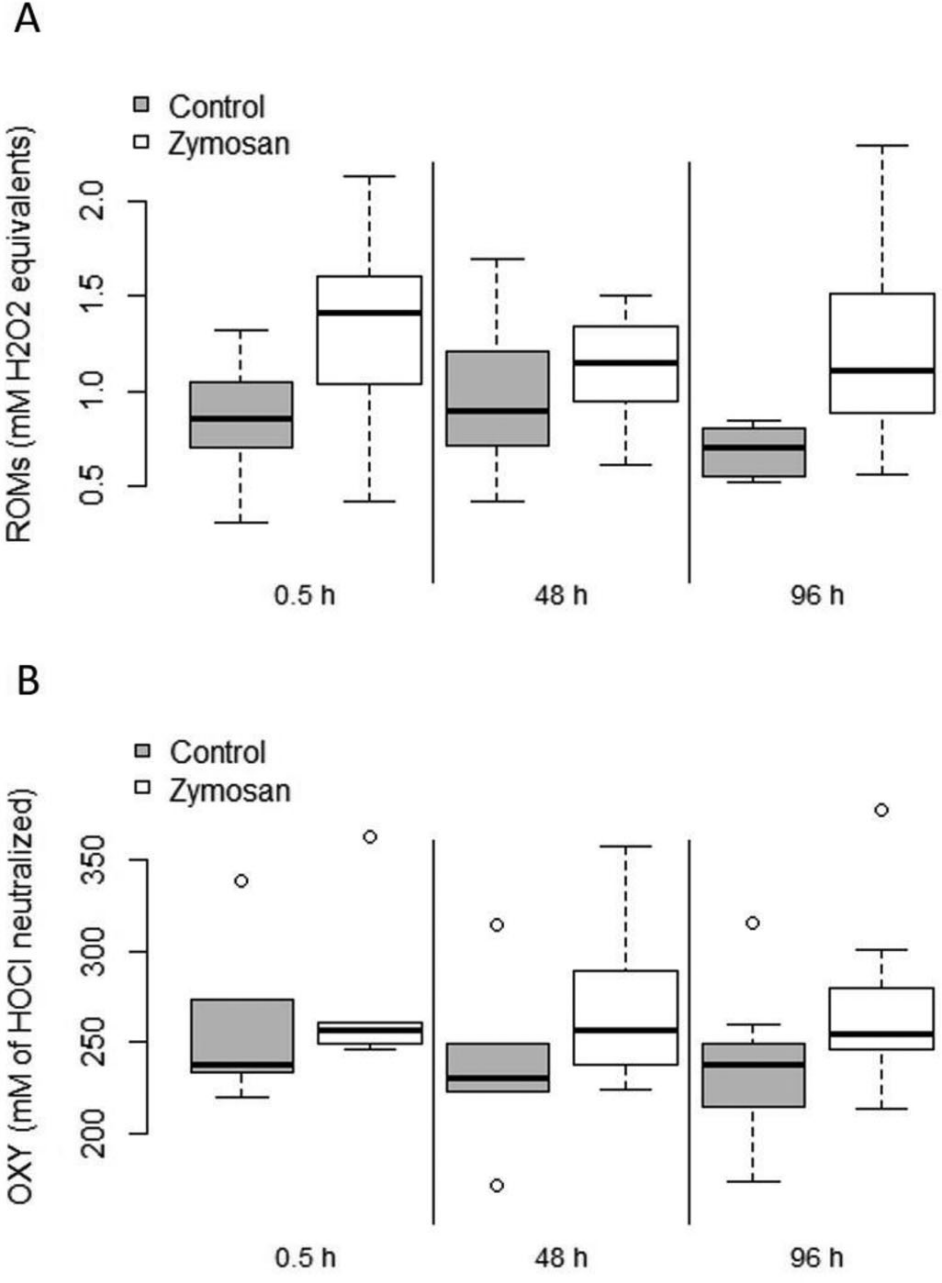
**Boxplot displaying the concentration of ROMs (A) and OXY (B) in the blood of the zymosan-treated bats (white) and bats of the control group (grey) on the three sampling days.** Upper and lower borders of boxes indicate first and third quantiles, respectively, whiskers represent maximum and minimum, solid horizontal line within boxes shows the median. Extreme values are shown as open circles.

**Fig. 7.**
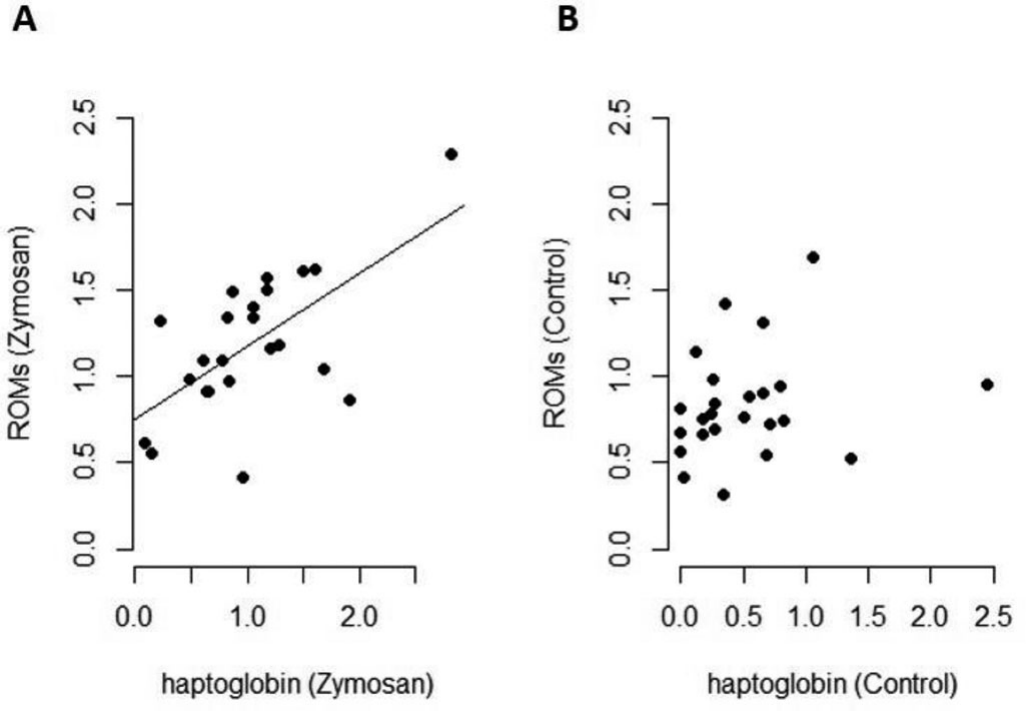
**Correlations between haptoglobin (mg/ml) and ROM levels (mMol H_2_O_2_ equivalents) of zymosan-treated bats (A) and control bats (B).** Haptoglobin correlated significantly with dROM in zymosan-treated bats (Pearson's rank correlation). One outlier was identified in zymosan-treated bats and tested for its influence on the correlation which turned out as negligible because the correlation after removing the outlier is still significant (cor=0.46, t=2.18, *P*=0.043).

We observed higher levels of reactive oxygen metabolites (ROMs) in zymosan-treated bats compared with bats in the control group (SS=1.138, *F*=7.74, *P*=0.017), but no difference among sampling days (SS=0.190, *F*=0.65, *P*=0.531). Also, the interaction between treatment group and sampling days was not significant for ROMs (SS=0.352, *F*=1.20, *P*=0.318) (Fig. 6A). There were also no differences in non-enzymatic antioxidant capacity (OXY), neither between the two treatment groups (SS=273.54, *F*=0.77, *P*=0.397), nor the sampling days (SS=653.53, *F*=0.92, *P*=0.414) nor the interaction between treatment group and days (SS=127.47, *F*=0.17, *P*=0.844) (Fig. 6B). The interaction of forearm and body mass had no influence on both response variables in the models (ROM: SS=0.003, *F*=0.02, *P*=0.896; OXY: SS=14.68, *F*=0.041, *P*=0.840). We observed a strong correlation between haptoglobin and ROM levels in bats of the zymosan-treated group (cor=0.630, t=3.71, *P*=0.001), which was not seen in bats of the control group (cor=0.216, t=1.04, *P*=0.312) (Fig. 7). One outlier was identified in zymosan-treated bats and tested for its influence on the correlation, which turned out as negligible because the correlation remained significant after removing this data point (cor=0.46, t=2.18, *P*=0.043).

### Circulating WBC

In both treatment groups we detected a significant increase in total WBC (SS=1.17E+13, *F*=3.06, *P*=0.047), lymphocytes (SS=2.37E+12, *F*=3.08, *P*=0.046) and monocytes (SS=1.18E+11, *F*=4.82, *P*=0.008) between sampling days. However, neither treatment group affiliation nor body condition of animals had a significant effect on WBC (Table 3, Fig. S1).

**Table 3.**
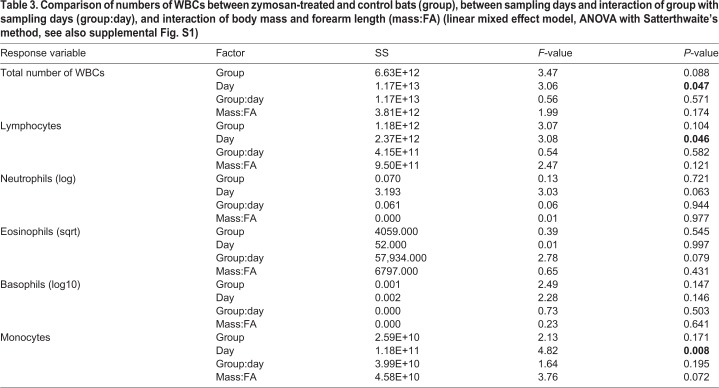
**Comparison of numbers of WBCs between zymosan-treated and control bats (group), between sampling days and interaction of group with sampling days (group:day), and interaction of body mass and forearm length (mass:FA) (linear mixed effect model, ANOVA with Satterthwaite's method, see also supplemental Fig. S1)**

## DISCUSSION

Using zymosan, a non-infectious fungal antigen, we challenged hibernating bats to induce an immune response and then followed several components of the acute phase response over a period of 4 days. We did not observe changes in torpor patterns, arousal frequencies or body temperatures between the two experimental groups. However, we noted an increase in gene expression of IL-1β, iNOS2 and TNFα for both zymosan-treated and control bats. In comparison to control bats, we only detected increasing haptoglobin levels in zymosan-treated bats, indicating an acute phase response. There was no difference in the levels of plasma oxidative status markers or WBC between groups. Also, individuals of both groups lost body mass to a similar extent. Interestingly, the functional response of the innate immune system seemed to be unlinked to torpor behavior or to the regulation of body temperature. Based on all these observations, we conclude that European hibernating bats such as *M. myotis* may have evolved a hibernation-adjusted immunological response to balance the trade-off between tolerance and resistance, and thus between mounting an immune reaction and its energetic costs.

### Torpor behavior, body temperatures and fever

During hibernation, immune functions of hypothermic bats are downregulated (reviewed by Bouma et al., 2010a). Our results suggest that European bats overcome this constraint by mounting only selected immune parameters such as haptoglobin during their regular arousals from torpor. Long-term data on body temperatures of hibernating European bats with infections are still lacking and thus it remains unclear if the thermoregulation of bats remains unaffected following a pathogen infection. However, over the short-term period of our experiment, we did not observe a strong effect of zymosan challenge on the torpor behavior of bats. In hibernating golden-mantled ground squirrels (*Spermophilus lateralis*) that were challenged with lipopolysaccharides (LPS), animals responded with fever during the arousal periods (Prendergast et al., 2002). Our zymosan-challenged *M. myotis* bats did not show any signs of a fever response, suggesting that bats seem to be prudent in the use of energy even when dealing with a fungal challenge in the torpid state. Interestingly, *Pd*-infected American bats exhibited fever bursts during arousals from hibernation (Mayberry et al., 2018) and bats surviving the *Pd*-epidemic in North America did not exhibit the ‘frequent arousal behavior’ of those that had died (Lilley et al., 2016). Thus, their behavioral pattern was more similar to that of European bat species. However, fever responses depend on the type of antigen and the level of infection or dosage (Bastos-Pereira et al., 2018); e.g. zymosan-challenged rats reacted with a fever response, but that response was dose-dependent with the dose being at least 3 mg/kg (Bastos-pereira et al., 2018). Hence, our zymosan dose (0.7 mg/kg) was likely high enough to induce an inflammatory response but might have been too low to induce a fever response in bats. Additionally, randomly appearing arousals of our bats might have been caused by ultrasonic emissions of the iButtons (Willis et al., 2009). We found slight decreases in body mass in both treatment groups (Fig. 1). However, the cost of mounting an immune response may be incurred at the level of torpid metabolic rate, which was not measured (McGuire et al., 2017).

### Inflammatory gene expressions

During the acute phase responses, inflammatory genes are usually upregulated and the inflammatory cells are activated together with the vascular system (Gruys et al., 2005). We found increases in the expression of all three genes during the course of the experiment (IL-1β, iNOS2 and TNFα), but they were not associated with the zymosan treatment. Thus, these increases might be rather due to the general reactivation of the immune system during the arousals and do not reflect a response to inflammation. Gene expression levels may change within hours (Breen et al., 2000; Fan et al., 1998; Jiménez-Ortega et al., 2009) and hence it could be that we were not able to detect differences between groups due to the time points of blood sampling (Jilma-Stohlawetz et al., 2017). Additionally, the interpretation of the gene expression results is hampered by the fact that we are lacking data on baseline levels. Additionally, measured RIN values suggest some decay of the RNA resulting from a 1-year storage of the RNA extracts. However, because all samples had been collected and extracted simultaneously and RNA extracts were stored at −80°C, a linear and uniform degradation rate of the isolated RNA can be assumed for all samples (Gallego Romero et al., 2014). Although these results have to be viewed cautiously, they fit into the picture of an adjusted, moderate immune response with genes becoming active, but not strongly expressed during arousal. Additionally, our observation confirms a recent study comparing *P. destructans* infected *Myotis lucifugus* and *M. myotis* showing an absence of immune gene expression in infected tissue of *M. myotis* (Lilley et al., 2019).

### Haptoglobin & oxidative stress

Contrasting the findings from a zymosan-challenge experiment on house sparrows (Coon et al., 2011), we measured higher levels of haptoglobin in zymosan-treated bats compared with control bats during hibernation. Haptoglobin is a hemoglobin-binding protein whose concentration is rapidly increased during acute phase responses in systemic infections (Cray et al., 2009). A study in bears has shown that haptoglobin levels are generally elevated in the late phase of hibernation (Mominoki et al., 2005), supporting our results for its important immunological function in hibernating mammals. The differential regulation of immunity-related proteins such as haptoglobin may be one adaptation during hibernation that allows mammals to remain in their hypometabolic and hypothermic state, while aiding in the maintenance of immune competence and resistance against infections and diseases (Chow et al., 2013). Acute phase proteins are part of the innate immune system and represent an early defense, which is immediately activated by inﬂammation (Cray et al., 2009) and effective for pathogen elimination (Bertaggia et al., 2014; Eaton et al., 1982; Langlois and Delanghe, 1996). Thus, the increased haptoglobin concentration in zymosan-treated bats reflects an inflammatory response to the fungal antigen. However, we could not directly show the link between haptoglobin increases and gene expression. Since haptoglobin can be mediated by different immune genes such as interleukin-6-type cytokines and is synergistically enhanced by glucocorticoids (Wang et al., 2001), we might have missed the detection due to our restriction on using only published primers (Kacprzyk et al., 2017).

Besides its function during an acute phase response, haptoglobin also prevents oxidative stress (Bertaggia et al., 2014; Gutteridge, 1987; Schaer et al., 2013; Tseng et al., 2004). We measured higher ROM levels in zymosan-treated bats, but these levels were already higher right from the first sampling and did not show further increases during the following days, which renders interpretation difficult. It might be that the concentration of ROMs is linked to haptoglobin and haptoglobin inhibited the increases due to its anti-oxidant effect (Sauerwein et al., 2005; van de Crommenacker et al., 2010; Corsetti et al., 2018). It is therefore conceivable that an increase in ROMs took place during the first arousal early in the experiment as a response against the antigen. During the following days any further increase may have been inhibited by haptoglobin. Consistent with this scenario, we found a significant correlation between ROMs and haptoglobin in zymosan-treated bats. However, the strength of the interaction between the inflammatory and some oxidative pathways is generally weak (Costantini et al. 2015; Sebastiano et al. 2018) and we are unsure if ROM concentrations can really increase during arousal events within a short period of 30 min (first measurement after pathogen challenge in our study in bats).

### Circulating WBCs

Neutrophils and macrophages are fundamentally important antifungal effector cells (Shoham and Levitz, 2005). During hibernation, when the body temperature is drastically lowered, the concentration of circulating WBCs is reduced by up to 90% (Bouma et al., 2010a). However, as shown in ground squirrels, leucocyte levels can be restored during an arousal within 1.5 h (Bouma et al., 2010b). In our experiment, the first blood sample was taken 30 min after the arousal had been induced. Hence, it is possible that the time point of measurement after arousal initiation was too early to detect the full increase in the number of leukocytes. However, we measured increases in numbers of certain leukocyte types during the course of the experiment, suggesting an accumulation of leucocytes with increasing numbers of arousals. Nevertheless, we did not find differences between zymosan-treated and control bats, neither in the total number of WBCs, nor in the numbers of specific WBC types. Therefore, we suggest that these increases are a regular response to the arousals (and thus reflect a general boost of ‘defense status’) but are not related to the immune challenge. We argue that during later arousals or even after hibernation, macrophages and neutrophils may even be further elevated in infected bats and that our results may not have covered the full immune response during the 5 days of the experimental period. Overall, an increase in WBCs is generally delayed during an infection compared with acute phase proteins such as haptoglobin (Cray et al., 2009).

## CONCLUSIONS

Our study suggests the presence of an adjusted immune function during hibernation as a strategy to retain immune competence while at a state of low metabolic activity. Both zymosan-treated and control bats lost approximately the same amount of body mass and did not differ in torpor patterns over the course of the experiment. However, zymosan bats mounted an acute phase response represented by haptoglobin increases but without increasing arousal frequencies and durations or maximal T_sk_ during arousal. This contrasts with previous studies suggesting that hibernating mammals have to increase their arousal frequency in order to spend a sufficient amount of time in euthermic conditions, or even show fever during arousals to mount an immune response (Bouma et al., 2010a; Carey et al., 2003; Luis and Hudson, 2006; Prendergast et al., 2002; Warnecke et al., 2012). Arousals, fever and general cellular immune responses are energetically costly as they act systemically. In comparison, the release of haptoglobin and pro-oxidants is much less costly yet still very effective against pathogens as they can be released by immune cells in different tissues. Hence, the adjustment of the immune response during hibernation could be characterized by investing only into those components which are both effective and fast. The efficacy of this immediate response could keep pathogens at bay until they can be fully cleared. The latter may then happen under euthermic conditions during later arousals or after hibernation in spring. From an energy saving point of view, a specialized first line defense based on the innate immune response, including an inflammatory response, is advantageous over a full immune response as long as the pathogen can be confined. In contrast, the activation of immune compounds such as WBCs and components of the adaptive immune system as shown in North American bats with white-nose syndrome may require the accelerated consumption of limited energy reserves without the subsequent benefits of pathogen clearance (Moore et al., 2013).

## MATERIALS AND METHODS

### Bat capture and housing

We captured sub-adult yearlings (14 males, two females) of greater mouse-eared bats (*M. myotis*) during autumn at a swarming site in Northern Bavaria (Germany) using two 10-m-long mist nets (Solida, Steinbach). Bats were transported to the field station of the Leibniz Institute for Zoo and Wildlife Research Berlin, where all individuals hibernated in individual boxes in a climate chamber, mimicking conditions in natural hibernacula [temperature 7–10°C and relative humidity 90–100% (Kulzer, 2008)]. Animal experiments were approved of by the Animal Experiments Committee of Brandenburg (permit no. 2347-43-2015) and the authority for conservation of protected species of Franconia, Germany (permit no. 55.1-8642.01-11/15).

### Immunological challenge

After 5 months of hibernation, bats were randomly assigned to either the zymosan treatment group (*N*=7 males, one female) or to the control group (*N*=7 males, one female). Bats of the zymosan treatment group received a subcutaneous injection of 0.7 mg/kg zymosan (Merck KGaA, previously Sigma-Aldrich, Darmstadt) dissolved in 100 µl sterile, isotonic phosphate buffered saline solution (PBS). The dose was derived from previous studies conducted on house sparrows (*Passer domesticus*) (Coon et al., 2011) and active *M. myotis* (Seltmann et al., unpublished results). The bats of the control group received 100 µl of PBS. The injections were conducted immediately after individuals had been taken out of hibernation chambers to ensure that body temperature was low in order to challenge bats in the state of hibernation before they aroused.

### Body temperature monitoring

To monitor body temperature, torpor and arousal patterns of bats, we used iButton^®^ temperature loggers (Thermochron iButton^®^, Maxim, San Jose, USA). Immediately after injection, the loggers (±0.5°C accuracy, factory programmed and calibrated) were attached to the dorsal skin of the interscapular region of the bats, right above to the injection area, by using waterproof medical sticking plaster (WUNDmed, Abenberg). We shaved the fur from this area in order to record the superficial skin temperature (T_sk_) as a proxy for the core body temperature (Currie et al., 2015; Warnecke et al., 2012). The metal surface of the iButtons had direct contact to the bat skin, without glue in between to avoid isolation effects and variations in contact with the body surface. Loggers were programmed before attachment (one measurement per 5 min interval) and the data was extracted after the experiment by using the 1-Wire^®^ system (Dallas Semiconductor, Dallas, USA).

### Blood sampling

We collected blood samples 30 min after the initial injection, placed all bats back into hibernation conditions and collected additional blood samples after 48 h and 96 h (Fig. 2). For the sampling procedure, we removed bats from the climate chamber and allowed the animals to arouse (30 min). We collected about 50 µl blood from the uropatagial vein of aroused bats using a sterile needle (20GX1″, WDT, Garbsen) and heparinized micro-hematocrit tubes (BRAND, Wertheim). We used 2.5 µl of each sample to prepare a blood smear and dissolved additional 5 µl in 100 µl Tuerk's solution (Merck, Darmstadt, Germany) for counting leukocytes. The remaining blood was centrifuged to separate plasma and blood pellet (buffy coat and erythrocytes) and the samples were stored for approximately 1 year at −80°C until further analysis.

### Expression of immune genes

RNA was extracted from blood pellets following the manual of the NucleoSpin^®^ RNA Blood extraction kit (Macherey & Nagel GmbH & Co. KG, Düren) with slight modification due to different type of sample used: we added 150 µl H_2_O to the blood pellet during the lysis step and added a second elution step to increase the RNA yields. The cDNA was synthesized using the ‘RevertAid H Minus First Strand cDNA Synthesis Kit’ (Thermo Fisher Scientific, Waltham, USA) according to the manufacturer's instructions. For the qPCR we used bat specific primers (Kacprzyk et al., 2017) for actin Beta (ACTB, housekeeping gene), IL-1β, TNFα, IL-10 and iNOS2 (Table 1). The qPCR was carried out on a CFX96 cycler (Bio-Rad, Munich, Germany) applying the ‘Real Time PCR iQ SYBR Green Supermix’ kit (Bio-Rad, Hercules, USA) according to the manufacturer's protocol. Prior to the analysis, we optimized the primer-specific annealing temperature (T_a_, Table 1). Reaction conditions were 3 min at 95°C; 40 cycles: 20 s at 95°C, 20 s at T_a_, 30 s at 72°C; melting curve analysis: 10 s at 95°C, 5 s at 60°C to 95°C in increments of 0.5°C. Quantitation cycle values (C_q_) determined during the quantitative real time PCR were used to express the levels of the immune genes relative to that of ACTB present as the relative expression ratio (rER): immune gene/ACTB. To assess RNA integrity, RIN values of the extracted total RNAs were determined on a 2200 TapeStation (Agilent) according to the manufacturer's instructions.

### Haptoglobin

Haptoglobin was measured following the standard procedure of the commercial ‘PHASE’™ Haptoglobin Assay (Tridelta, Maynooth, Ireland) which was previously used in other bat species (Costantini et al. 2019). As an acute phase protein, haptoglobin reduces oxidative damage by binding hemoglobin released during hemolysis, has immune-modulatory effects and inhibits bacterial growth. Haptoglobin binds to hemoglobin and maintains its peroxidase activity at a low pH. The measured peroxidase activity of hemoglobin is directly proportional to the amount of haptoglobin in the sample. After we diluted the plasma samples (1:4) with PBS, we added hemoglobin. Haptoglobin concentrations (mg/ml) were calculated according to the standard curve on each plate.

### Oxidative status

We measured reactive oxygen metabolites (ROMs) and non-enzymatic antioxidant capacity (OXY), two markers of oxidative status known to be associated with immune responses (e.g. Costantini and Møller, 2009; Schneeberger et al., 2013a,b). We determined the ROMs using the d-ROMs test (Diacron, Grosseto, Italy) - a test that quantifies the number of damaged molecules generated early in the oxidative cascade (e.g. organic hydroperoxides, endoperoxides). We diluted 4 μl of plasma in 200 μl of a solution containing 0.01 M acetic acid/sodium acetate buffer (pH 4.8) and N,N-diethyl-p-phenylenediamine as chromogen. This mixture was incubated for 75 min at 37°C. Analyses were performed in duplicates on the same plate (CV=4.8%). After incubation, absorbance was measured at 505 nm with a spectrophotometer (μQuant Microplate Spectrophotometer, Bio-Tek). ROM concentrations were calculated by plotting values onto a calibration curve obtained by measuring the absorbance of a standard solution. Values were expressed in mM of H_2_O_2_ equivalents. The OXY-adsorbent test (Diacron, Grosseto, Italy) was used to quantify the non-enzymatic antioxidant capacity of plasma. This test is based on an *in vitro* reaction of non-enzymatic antioxidants (e.g. both lipophilic and hydrophilic antioxidants, such as vitamins, protein thiols, etc.) that occur in a biological matrix (e.g. plasma, hemolysate, tissue homogenate) with hypochloric acid (HOCl). We carried out our analyses using 200 µl of diluted plasma (2 µl of plasma 1:100 with distilled water). To the diluted plasma we added 200 μl of HOCl followed by incubation at 37°C for 10 min. Analyses were carried out in duplicates on the same plate (CV=4.4%). After incubation, absorbance was also measured at 505 nm. We calculated the antioxidant capacity using a reference standard. Values were expressed as mM of HOCl neutralized.

### WBC counts

Total numbers of WBCs were calculated by using the cell counts obtained from diluted blood on a Neubauer chamber using the formula: number of cells in the fields×10,000/number of fields×dilution=WBCs/ml (Bastidas, 2014; Weise et al., 2017). The number of WBCs per different cell type was determined by counting the different WBC types among 100 immune cells on a May-Grünwald-Giemsa stained blood smear under a microscope at 1000× magnification (Blumenreich, 1990; Schneeberger et al., 2013b; Seltmann et al., 2017). The concentrations of the different WBC types (lymphocytes, neutrophils, eosinophils, monocytes, basophils) were then determined by calculating the proportion of each WBC type in the total WBC concentration. All WBC counts are reported in number of cells per 1 µl blood.

### Calculations and statistical analysis

Torpor and arousal events were recognized by changes of skin temperature (T_sk_) recorded at 5 min intervals. Arousals were determined by a T_sk_ increase of at least 10°C and lasting at least 20 min (Lilley et al., 2016). Torpor was determined by T_sk_ falling below 11°C for at least 2 h. Artificial arousals due to bat handling (inoculation, blood sampling) and natural arousals were analyzed separately.

All statistics were done in R (version 3.6.0). Data from all response variables was tested for normal distribution (Shapiro-Wilk test and Lillie test). Non-normal distributed data was transformed per square root (sqrt), natural logarithm (log_e_) and 10 (log_10_) into normal distributions (for details see Results). The number of arousals could not be transformed into normal distribution and was analyzed by Kruskal–Wallis rank sum test. Mean body temperature during torpor, mean body temperature during arousal and maximum temperatures during arousal were obtained from individual T_sk_ records. Differences between zymosan-treated and control bats were calculated by ANOVA. Body mass data, WBC counts, concentrations of haptoglobin, ROMs, OXY, and rER were analyzed by linear mixed-effects models (lmer) fitted by the Restricted Maximum Likelihood (REML) method from the R package ‘lmerTest’ (Kuznetsova et al., 2017). As factors we here included treatment groups, sampling days, interaction between treatment group and sampling days, and individual body mass and forearm length. The individual was included as random factor. For the body mass lmer-analysis we set treatment group, sampling days, and interaction between them as factors, forearm length as covariate and individual as random effect. All fitted models were analyzed by ANOVA and stochastic dominances among factors were calculated by *t*-tests using Satterthwaite's method (Kuznetsova et al., 2017). *P*-values were adjusted for multiple comparisons by using Tukey's method in the ‘emmeans’ package (Lenth et al., 2019). Because haptoglobin may correlate with ROM (Corsetti et al., 2018; Sauerwein et al., 2005; van de Crommenacker et al., 2010), the relationship was calculated by using the Pearson's rank correlation for each treatment group.

## Supplementary Material

Supplementary information
