# Supplementary material for: Immune response of hibernating European bats to a fungal challenge
Source: Biol Open. 2019 Oct 24;8(10):bio046078. doi: 10.1242/bio.046078 (PMC6826279; doi:10.1242/bio.046078)
Supplement: Supplementary information [file biolopen-8-046078-s1.pdf]

Supplementary material

Table S1: Mean time span (duration) from end of torpor state until the temperature had increased by at least 10°C ($\Delta T_{arousal}$) and until reaching the maximum arousal temperature (max $T_{arousal}$) per treatment group.

group	arousal type	level	mean duration (min)
zymosan-treated	artificial	$\Delta T_{arousal}$ >10°C	20.94
control	artificial	$\Delta T_{arousal}$ >10°C	21.56
zymosan-treated	natural	$\Delta T_{arousal}$ >10°C	21.00
control	natural	$\Delta T_{arousal}$ >10°C	21.25
zymosan-treated	artificial	max $T_{arousal}$	36.56
control	artificial	max $T_{arousal}$	35.33
zymosan-treated	natural	max $T_{arousal}$	24.25
control	natural	max $T_{arousal}$	24.63

Table S2. RIN-values of total RNAs extracted from blood clot samples of 16 *Myotis myotis (Mmyo)*. The RIN-values (RNA integrity values) were measured in order to assess the state of the extracted RNA. The values were determined using the 2200 TapeStation (Agilent) and the RNA ScreenTape. / - no value could be measured. In comparison, fresh *Mmyo*-tissue had a RIN-Value=8.

0.5 h after inoculation		48 h after inoculation		96 h after inoculation	
Sample	RIN-value	Sample	RIN-value	Sample	RIN-value
Mmyo1	4.3	Mmyo1	3.3	Mmyo1	3.4
Mmyo2	4	Mmyo2	3.5	Mmyo2	3.3
Mmyo3	/	Mmyo3	3.6	Mmyo3	2.4
Mmyo4	3.3	Mmyo4	4.3	Mmyo4	3.9
Mmyo5	3.5	Mmyo5	4.2	Mmyo5	4.1
Mmyo6	3.9	Mmyo6	4.8	Mmyo6	4.1
Mmyo7	3.7	Mmyo7	3.9	Mmyo7	3.6
Mmyo8	3.9	Mmyo8	3.8	Mmyo8	1
Mmyo9	3.6	Mmyo9	4.2	Mmyo9	4.5
Mmyo10	3.8	Mmyo10	3.6	Mmyo10	4
Mmyo11	3.8	Mmyo11	3.6	Mmyo11	3.1
Mmyo12	4.8	Mmyo12	4.8	Mmyo12	5.5
Mmyo13	5.1	Mmyo13	6.6	Mmyo13	5.9
Mmyo14	3.8	Mmyo14	2.6	Mmyo14	/
Mmyo15	3	Mmyo15	3.3	Mmyo15	3.9
Mmyo16	4.9	Mmyo16	3.8	Mmyo16	3.9

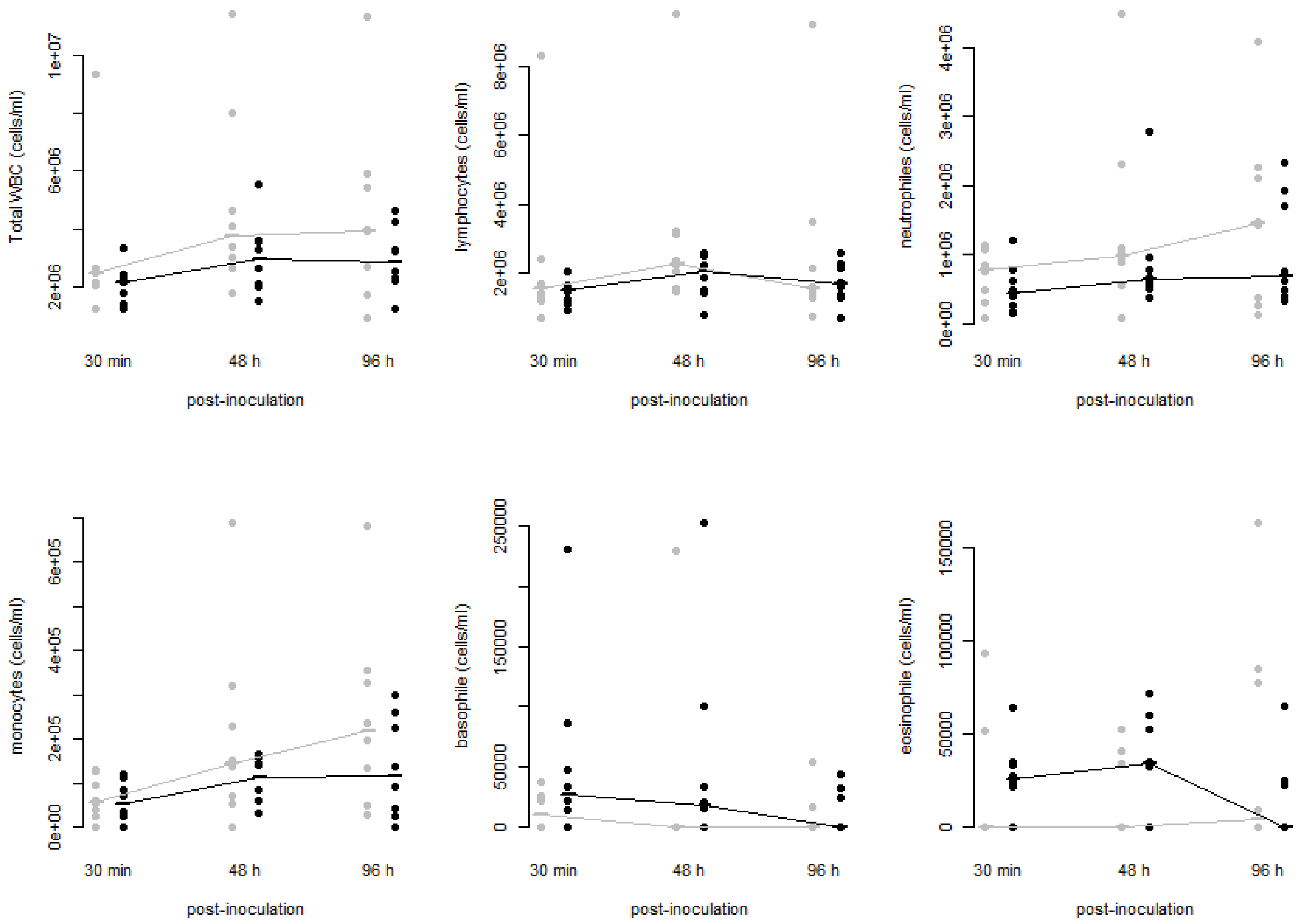

Figure S1. Concentrations (in cells / µl blood) of different white blood cells (WBC) in zymosan-treated (black) and control bats (grey)

Biology Open • Supplementary information
